# Pharmacological Polarization of Tumor‐Associated Macrophages Toward a CXCL9 Antitumor Phenotype

**DOI:** 10.1002/advs.202309026

**Published:** 2024-02-11

**Authors:** Noah Enbergs, Elias A. Halabi, Anne‐Gaëlle Goubet, Kelton Schleyer, Ina R. Fredrich, Rainer H. Kohler, Christopher S. Garris, Mikaël J. Pittet, Ralph Weissleder

**Affiliations:** ^1^ Center for Systems Biology Massachusetts General Hospital 185 Cambridge St, CPZN 5206 Boston MA 02114 USA; ^2^ Department of Pathology and Immunology University of Geneva Geneva 1211 Switzerland; ^3^ AGORA Cancer Center Swiss Cancer Center Leman Lausanne 1011 Switzerland; ^4^ Ludwig Institute for Cancer Research Lausanne 1005 Switzerland; ^5^ Department of Systems Biology Harvard Medical School 200 Longwood Ave Boston MA 02115 USA

**Keywords:** CXCL9, IFNg, macrophage, nanoparticles, PARP7, polarization, STING

## Abstract

Tumor‐associated macrophages (TAM) are a diverse population of myeloid cells that are often abundant and immunosuppressive in human cancers. CXCL9^Hi^ TAM has recently been described to have an antitumor phenotype and is linked to immune checkpoint response. Despite the emerging understanding of the unique antitumor TAM phenotype, there is a lack of TAM‐specific therapeutics to exploit this new biological understanding. Here, the discovery and characterization of multiple small‐molecule enhancers of chemokine ligand 9 (CXCL9) and their targeted delivery in a TAM‐avid systemic nanoformulation is reported. With this strategy, it is efficient encapsulation and release of multiple drug loads that can efficiently induce CXCL9 expression in macrophages, both in vitro and in vivo in a mouse tumor model. These observations provide a window into the molecular features that define TAM‐specific states, an insight a novel therapeutic anticancer approach is used to discover.

## Graphical abstract

A 17 nm carbohydrate drug delivery system is used to shuttle a triple combination of a PARP7 inhibitor, a TLR7/8 agonist, and a STING agonist into tumor‐associated macrophages via systemic administration. This results in high CXCL9 induction in vitro and in vivo and antitumor efficacy.

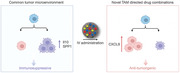

## 1 Introduction

The tumor microenvironment (TME) is dynamic^[^ ^1^, ^2^, ^3^ ^]^ and often contains large numbers of myeloid cells, especially tumor‐associated macrophages (TAM). In most human cancers, the majority of TAM is immunosuppressive. However, several different antitumor TAM phenotypes have recently been identified by single‐cell RNA sequencing, spatial biology methods, and flow cytometry. These antitumor phenotypes include TAM subsets that i) produce the chemokine ligand 9 (CXCL9),^[^ ^4^, ^5^, ^6^, ^7^, ^8^ ^]^ ii) produce interleukin 12 (IL12)^[^ ^9^, ^10^, ^11^ ^]^ or iii) lack secreted phosphoprotein 1 (SPP1).^[^ ^4^, ^12^ ^]^ Most recent research has shown that macrophage polarity, defined by the expression of two inversely regulated proteins, CXCL9 and SPP1 (abbreviated as “C/S” phenotype), was much more clinically predictive than the conventional M1 (e.g., defined by ITGAX, CD80) and M2 (e.g., defined by CD163, MRC1, Arg1) classification.^[^ ^4^ ^]^ This has re‐framed our understanding of antitumor TAM phenotypes, motivating the development of new therapeutic approaches to polarize macrophages.

IL12, one of the above‐mentioned cytokines, has previously been modulated pharmacologically.^[^ ^9^, ^10^, ^11^, ^13^ ^]^ Unfortunately, the prior formulations showed no CXCL9 up‐regulation.^[^ ^14^ ^]^ The chemokine CXCL9 promotes antitumor lymphocytic infiltration in solid tumors via its receptor CXCR3. Its production in TAM is believed to be mostly regulated by interferon‐gamma (IFNg) stimulation and signal transducer and activator of transcription 1 (STAT1) pathway^[^ ^15^, ^16^ ^]^ but possibly also by other pathways (e.g., Toll‐Like Receptor (TLR) via Nuclear Factor kΒ (NFkB),^[^ ^17^ ^]^ Stimulator of Interferon Genes (STING),^[^ ^18^ ^]^ and Poly‐ADP‐ribose polymerase 7 (PARP7), a member of the monoPARP family, key regulator of stress response and IFNg secretion).^[^ ^19^ ^]^ Additional potential regulators of CXCL9 are emerging (e.g., Mcidas, Spata22, Eed, Irf1, LIF, EZH2) but not all are currently druggable, and their expression is not unique to TAM,^[^ ^20^, ^21^, ^22^ ^]^ making the identification of small‐molecules capable of CXCL9 induction in TAM difficult and currently challenging.

The first goal of the current project was to determine whether small‐molecules could be used to polarize macrophages toward the newly defined CXCL9^Hi^ phenotype. A secondary goal was to formulate any such hits into a TAM‐targeting preparation to enhance treatment efficacy and reduce systemic toxicity. Therefore, we performed a screen of known small‐molecules targeting some of the above pathways to determine whether they induce CXCL9 production in bone marrow‐derived macrophages (BMDM). We reasoned that a scalable, in vitro, approach for testing CXCL9 induction in macrophages would lead to a therapeutic combination. We hypothesized that dual and triple combinations of different small‐molecules could synergize, prevent compensatory resistance mechanisms, and then be formulated into a single TAM‐avid drug delivery system. We discovered a triple combination that indeed increased CXCL9 production by an order of magnitude above traditional CXCL9‐inducing stimuli and much more efficiently than IFNg alone.

These findings are significant as they offer a promising avenue for developing novel therapeutic strategies targeting TAM. The ability to augment CXCL9 production in TAM can potentially increase lymphocytic infiltration, which drives antitumor immunity. This approach may hold promise for advancing cancer immunotherapy and improving treatment outcomes for cancer.

## 2 Results

### 2.1 Optical Screens in Freshly Isolated Target Cells

**Figure 1:**
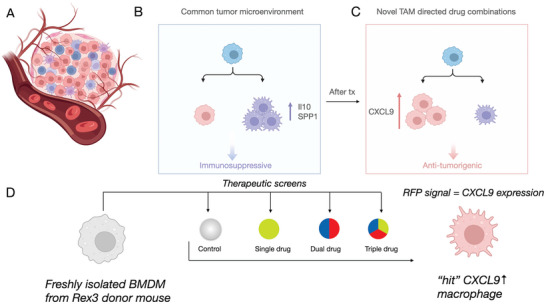
Overview of myeloid cell screening approach. A) TAM are bone marrow‐derived, abundant in many cancers, and mostly immunosuppressive and thus pro‐tumorigenic. B, C) This phenotype is driven primarily by IL10 and hypoxia (SPP1) signaling. We hypothesized that it should be possible to polarize macrophages to an antitumorigenic phenotype by increasing CXCL9 signaling.^[^ ^4^ ^]^ Yet, no effective pharmaceutical strategies have emerged to do this effectively while retaining TAM specificity. D) In this research, we used BMDM from Rex3 reporter mice (expressing CXCL9‐RFP) to screen for small‐molecules and combinations that could induce CXCL9 in myeloid cells. Top hits from the screen were then encapsulated into TAM‐avid nanoparticles to elicit a CXCL9 phenotype in vivo.

Pharmacological modulation of TAM in vivo has been challenging due to several reasons: first, the lack of cost‐effective methods to combinatorially screen drugs that can polarize TAM; second, the still limited knowledge of key regulators of TAM programming; and third, the availability of efficient and selective delivery vehicles with high drug payloads. To address these challenges, we have developed optically resolved screening approaches that more efficiently and rapidly identify therapeutic combinations that induce CXCL9 and other interferon‐stimulated genes (ISG) (**Figure** **1**). In this study, we performed the screening in primary isolated bone marrow‐derived cells (BMDC) from CXCL9 red fluorescent protein (RFP) and CXCL10 blue fluorescent protein (BFP) reporter mice to identify potential small‐molecule compound hits capable of inducing RFP expression. Freshly obtained BMDC from these mice were cultured with macrophage colony‐stimulating factor (M‐CSF) for 7 days, at which time they typically have low levels of baseline CXCL9 expression. Upon the addition of potential modulators fluorescence microscopy and flow cytometry can be used to measure increases in RFP (Figure 1).

### 2.2 Creation of a Mini‐Library for Screening

**Figure 2:**
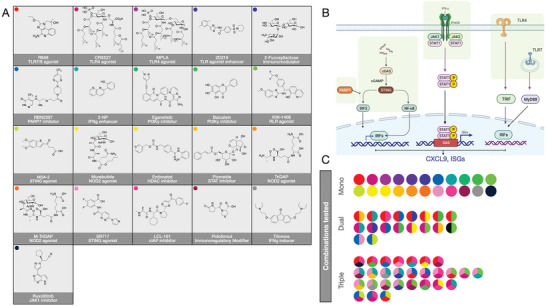
Small‐molecule compounds tested. A) Summary of the chemical structure of the 21 small‐molecule compounds tested. The colored dots represent individual compounds for identification across figures. B) Different classes of compounds are considered according to the current understanding of CXCL9 regulation in macrophages. C) Summary of single compound screens, dual compound screens, and triple compound screens. The colors of the dots represent the molecular structures shown in panel A.

There is an emerging realization that combination therapies are needed to: i) improve treatment efficacy, ii) lower the dose of single immune‐stimulatory agonists, and iii) circumvent immune cell resistance mechanisms. Based on the hypothesis that certain small‐molecule combinations may indeed affect distinct cellular programs when delivered specifically to TAM, we curated a small collection of drugs (**Figure** **2**). We focused primarily on known modulators of several major pathways (IFNg, NFkB, TLR, STAT1, and interleukin 10 (IL10). As no direct single agonist of IFNg signaling nor CXCL9 has been reported, we adapted our imaging readout approach to measure dozens of therapeutic combinations at varying dose levels for their ability to boost CXCL9 in macrophages. The selection of these compounds was largely driven by the current understanding of TAM signaling (Figure 2) and the potential of a given compound to be useable clinically. Our collections contained 21 individual small‐molecules and another ≈20 combinations, resulting in screens with ≈40 different drug combinations. Figures S2 and S3 (Supporting Information) summarize the synthesis and characterization of some of the compounds in our screen that are not commercially available.

### 2.3 Screening Identifies Drug Combinations that Induce CXCL9

**Figure 3:**
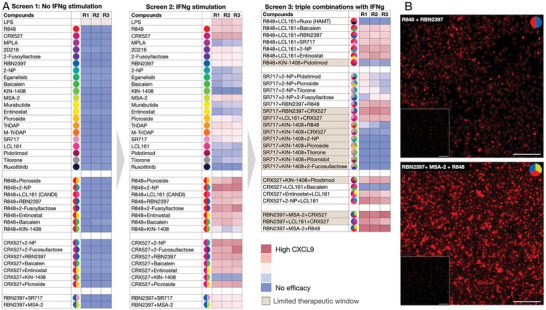
Screening results. Three separate screens were performed, each one informing the design of the next screen. A) Screen 1 was performed without IFNg stimulation of BMDM and explored CXCL9 TAM expression after single or dual agent exposure, as shown. Note the lack of efficacy of any of the compounds, indicating that baseline levels of IFNg are likely required for pharmacological CXCL9 induction. Screen 2 repeated the same screen but with baseline stimulation of IFNg. Note that some of the dual combinations yielded elevated levels of CXCL9. Screen 3 largely explored triple drug combinations with IFNg stimulation. Note the highest CXCL9 expression of combinations involving RBN2397, CRX527, R848, and/or MSA‐2. In parallel experiments, drug toxicity was determined. Drugs or combinations with a limited therapeutic window are shown in light brown. Given these results, the top hit emerging from the screen was the triple combination of RBN2397+MSA‐2+R848 which was then formulated into the CANDI400 formulation shown in subsequent figures. B. Representative images (550 nm channel, RFP) obtained from screens 2 (dual compound R848+RBN2397) and screen 3 (Bottom triple compound RBN2397+MSA‐2+R848). The black inserts represent the negative controls without drugs and IFNg stimulation alone. Scale bar 200 µm. See Figure S7 (Supporting Information) for other compounds and cytokines.

**Figure** **3** summarizes the primary screening results. All screenings were carried out in triplicates, yielding consistent and reproducible results with minimal variation. The first screening, performed as a control, examined whether any single compound or dual combination thereof could induce CXCL9 production in nonstimulated baseline macrophages. As expected, the screening did not identify any significant hits capable of elevating CXCL9 expression in vitro.

The second screening paralleled the first, yet it incorporated baseline IFNg stimulation to mimic native cytokine exposure within the tumor environment, if any. IFNg is primarily secreted by T‐cells and NK cells, instigating macrophage and dendritic cell responses via the interferon‐gamma receptor (IFNGR) and JAK/STAT pathways. This time, most single agents induced a mild increase in CXCL9 response, and certain dual combinations induced a moderate increase. However, specific pairings, particularly those including the RIG‐1‐like receptor agonist KIN‐1408, resulted in cellular toxicity and did not induce CXCL9.

Based on these results, we performed a third screening using triple drug combinations to determine whether CXCL9 expression could be further enhanced. Certain combinations with KIN‐1408 and CRX527 (an LPS mimetic) were associated with toxicity at the doses applied, leading to their exclusion from subsequent testing. The screening identified several triple combinations that achieved exceptionally high CXCL9 expression. The key finding resulting from the third screen was the combination consisting of RBN2397 (a PARP7 inhibitor), MSA‐2 (a STING agonist), and R848 (a TLR7/8 agonist) capable of increasing CXCL9 expression ∼8 fold. Interestingly, the expression of IL12 was also enhanced by this triple combination (Figure 3). Subsequent experiments were then performed to incorporate this potent combination into a TAM‐avid nanotherapeutic formulation (CANDI400).

### 2.4 CANDI400 Formulation and Efficacy

We had previously developed a cyclodextrin‐adjuvant nanoparticle‐drug delivery system (CANDI) that accumulates avidly in TAM after systemic injection and enables highly effective, multitarget, and pathway modulation inside TAM and dendritic cells.^[^ ^9^, ^14^ ^]^ Specifically, versions of this delivery platform had primarily been used to induce IL12 in TAM.^[^ ^14^ ^]^ Unfortunately, the prior drug combination “HAMT” (LCL161, R848, and Ruxolitinib) had no effect on cellular CXCL9 expression (Figure 3).^[^ ^14^ ^]^

We first validated the propensity of our hit drug combination to form strong complexes with the bisuccinyl cyclodextrin (sbCD) monomers of CANDI nanoparticle. Initially, we looked for changes in chemical shifts and equimolar complexation in nuclear magnetic resonance (NMR) titration experiments with R848, RBN2397, and MSA‐2 individually. These payloads were mixed in a D_2_O solution containing a fixed concentration of the host, sbCD (Figure S4, Supporting Information).^[^ ^14^ ^]^ From these experiments, we concluded that R848 had a very high binding affinity toward sbCD and potentially multiple binding orientations which favored its high solubility. RBN2397 had optimal complexation at a three‐host per guest complexation ratio. Potentially accounting for two aromatic and one aliphatic binding moieties (Figure S4, Supporting Information). Throughout all measured conditions, however, MSA‐2 showed very poor affinity to sbCD and remained a stable turbid suspension regardless of the presence or concentration of sbCD, hindering our NMR comparative studies in D_2_O.

For this reason, we opted to design a more lipophilic prodrug of MSA‐2 with enhanced inclusion complexation ability with sbCD, termed MSA‐2p. The synthesis of MSA‐2p was achieved in moderate yields activating MSA‐2 with 1‐ethyl‐3‐(3‐dimethylaminopropyl)carbodiimide (EDC). Using an anhydrous mixture of acetonitrile and methanol resulted in the highest yields (56%). MSA‐2p was the main product from this reaction (>85% conversion) and interestingly, the structure differed significantly from the other MSA‐2 prodrug analogs. MSA‐2p is a peculiar cyclic lactone locked in a chiral conformation as shown on the aliphatic region of the ^1^H‐NMR spectra (Figure S3, Supporting Information).

**Figure 4:**
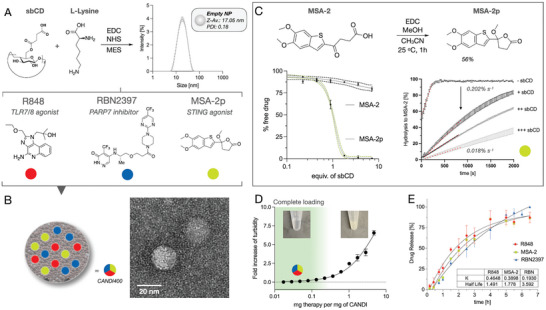
Formulation of small‐molecule hits into TAM‐avid nanoparticle (CANDI400). A. Synthetic strategy to form ≈17 nm nanoparticles consisting of sbCD cross‐linked by L‐lysine linkers via EDC/NHS chemistry. B. Transmission electron microscopy images revealed that the nanoparticles retained a spherical form and were able to carry the proposed three small‐molecule payloads. C. Synthesis of MSA‐2p from MSA‐2. The left bottom graph indicates the percent of free/bound drug to sbCD, determined as turbidity value (measured at 550 nm, average of 500 s time‐lapse, N = 3). Bulk studies using MSA‐2p in buffer (pH 7.4) showed that complexation to sbCD leads to a significant reduction in the hydrolysis rates of MSA‐2p (right). D. Turbidity assay to measure loading and stability of the CANDI400 formulation assessed by an increase in absorbance at 550 nm. We determined the loading capacity for CANDI400 to be ≈0.15 mg of payloads per mg of CANDI nanoparticle. E. Release kinetics of the triple small‐molecule combo from CANDI400 using a porous membrane (3 kDa) in PBS (1×) at 37 °C. The release rates for R848, MSA‐2, and RBN2397 are depicted as the dissociation rates (k_off_) and complex half‐life (t_1/2_), respectively determined in a 7 h time lapse (see Figure S6, Supporting Information, N = 3).

Solution experiments with MSA‐2p showed a fast decrease in turbidity when quasi‐equimolar concentrations of sbCD were present, indicating a strong affinity between MSA‐2p and sbCD. In the absence of the host, MSA‐2p had low water solubility (<3.6 mg mL^−1^, **Figure** **4**). We then examined the hydrolysis rate of MSA‐2p to MSA‐2. We used the spectrally unique 325 nm absorbance peak to quantify the formation of MSA‐2 in buffered solutions. We studied the hydrolysis rates of MSA‐2p from absorbance scans acquired at equilibrium and assessed that the complexation of MSA‐2p to sbCD had a significant effect on the hydrolysis rates. When dissolved in phosphate‐buffered saline (PBS), regardless of pH, MSA‐2p exhibited very fast hydrolysis (0.22% s^−1^). Adding an increasing amount of sbCD to six equivalents dropped the hydrolysis rate to (0.018% s^−1^ ≈11.8‐fold). These results indicate that the strong binding with sbCD hinders the nucleophilic attack of the water molecule, suggesting that the inclusion complexation inserts around the cyclic chiral lactone (details on the proposed hydrolysis mechanism can be found in Figure S5, Supporting Information).

In the next set of experiments, we performed typical nanoparticle characterization experiments of the triple‐loaded CANDI400 formulation (Figure S6, Supporting Information). Upon loading, the nanoparticle size was 19.8 ± 1.1 nm, and the zeta potential was −6.15 mV. Transmission electron microscopy revealed spherical polymeric structures with matching size distribution to our dynamic light scattering (DLS) measurements. We determined the drug loading capacity to be around 0.15 mg of drug combo (R848, RBN2397, and MSA‐2p) per mg of particle before it reaches saturation. In a closed‐dialysis set‐up mimicking physiological conditions, we determined the release rates for each drug in the CANDI400 formulation. The release of each released drug was quantified simultaneously using an optimized liquid chromatography method coupled to mass spectrometry (LCMS), which resulted in similar dissociation rates k_off_ (0.46, 0.39, and 0.19 h^−1^ for R848, MSA‐2, and RBN2397) and a complexation half‐life of t_1/2_ = 1.5, 1.8 and 3.6 h respectively (Figure 4 and Figure S6, Supporting Information).

**Figure 5:**
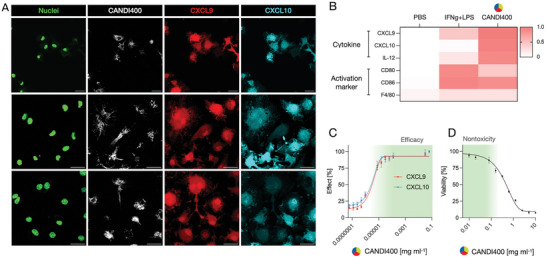
Cellular properties of CANDI400. A. BMDM obtained from Rex3 reporter mice were incubated with CANDI400 for 24 h and then observed by microscopy. Cellular nuclei were stained with SYTO™ 11 Green (green, 473 nm channel), CANDI400 was revealed by labeling the NP with AF647 (white, 633 nm channel). CXCL9 (red) and CXCL10 (cyan) were imaged by endogenous fluorescent protein expression (RFP, 559 nm channel and BFP, 405 nm channel respectively). Note the high CXCL9 levels in all cells containing CANDI400. Scale bar: 20 µm. B. Comparative measurements of key cytokines and TAM activation makers using flow cytometry. Note the high CXCL9 expression with CANDI400, even higher than with IFNg and LPS stimulation of cells. C. Dose‐response curve with increasing concentration of CANDI400, revealing EC_50_ values of 3.4 ng mL^−1^ for CXCL9 and 3.2 ng mL^−1^ for CXCL10, respectively. D. Cellular toxicity using the MTT assay. Note the lack of toxicity at drug concentrations <0.1 mg mL^−1^. C and D were used to estimate drug concentrations for in vivo experiments (green‐shaded area).

A dose‐response curve was obtained with BMDM from CXCL9‐RFP mice, yielding an effective EC_50_ of 3.3 ng ml^−1^. As shown in **Figure** **5**, there was uniform CANDI400 uptake and concomitant CXCL9 production in BMDM. Additional cytokine screening experiments were conducted (Figure S1, Supporting Information) to ascertain that cellular cytokine production was due to the CANDI payload and not the nanoparticle drug carrier itself. At this dose, there was no discernible cellular toxicity with CANDI400. These results confirmed the efficacy of the triple combination nanoparticle to increase CXCL9 production. Subsequent experiments were therefore conducted to show in vivo efficacy.

### 2.5 Intravital Imaging Reveals Drug Action in the Tumor Microenvironment and Antitumor Efficacy

**Figure 6:**
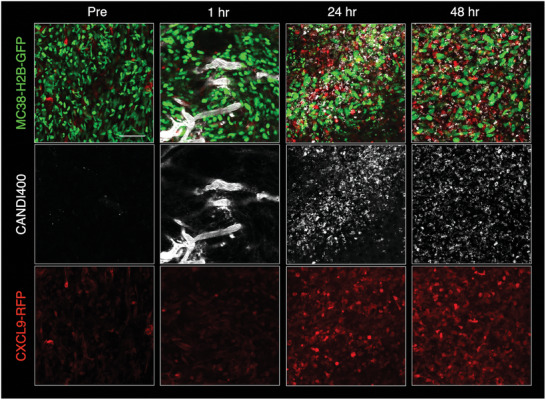
Intravital microscopy of CXCL9 induction in a tumor mouse model. A. Serial imaging of the TME in the colorectal MC38‐H2B‐GFP tumor model. Eight days after tumor implantation, CANDI400 was administered by tail vein, and serial repeat imaging was performed. CANDI400 was labeled with AF647 and contained the triple small‐molecule cocktail (RBN2397, MSA‐2p, and R848) to induce CXCL9 in myeloid cells in the TME. The top row (scale bars 50 µm) shows MC38‐H2B‐GFP tumor cells (green, 488 nm channel), the middle row CANDI400^AF647^ (white, 647 nm channel), and the lower row CXCL9‐RFP (red, 550 nm channel). Immediately after intravenous injection of CANDI400, the nanotherapeutic drug is largely confined to vessels. Within several hours, the CANDI is later taken up by TAM. Note the high CXCL9 induction within 24 h after systemic CANDI400 administration. See Figure S8 (Supporting Information) for high‐resolution images.

We first determined the vascular half‐life of CANDI400 by serial imaging of the microvasculature in the mouse ear (Figure S9, Supporting Information). This showed a vascular half‐life of approximately 2.3 h. Cellular uptake could be identified as early as 1 h but was more pronounced by 4 h after IV administration. To determine whether the CANDI400 formulation indeed accumulated within TAM in vivo following systemic administration and elicited CXCL9 expression, we performed intravital microscopic imaging in live mice. CXCL9‐RFP transgenic mice were implanted with dorsal window chambers into which MC38‐GFP tumors were grown. After 8 to 10 days, we performed serial microscopic examinations of the TME both before and after intravenous systemic administration of CANDI400 labeled with AF647 (**Figure** **6** and Figure S8, Supporting Information). Baseline expression of CXCL9 pre‐treatment was very low, with sparse cellular expression within the TME. Remarkably, within 24 to 48 h postinfusion of CANDI400, we observed a considerable induction of CXCL9 throughout the tumor. Using a fluorescent analog of our particles labeled with AF647, we confirmed that these CXCL9‐expressing cells had incorporated the nanoformulation (Figure 6 and Figure S8, Supporting Information).

To determine whether these changes translate to antitumor efficacy, we performed tumor growth experiments in mouse models (Figure S10, Supporting Information). We observed remarkable efficacy in the MC38 tumor model, with all tumors disappearing after two systemic administrations of CANDI400. 60 days after tumor inoculation, all mice that received CANDI400 were still alive, in contrast to a median survival time of 28 days in the control group. Figure S11 (Supporting Information) summarizes our understanding of how this therapeutic combination works in vivo. These results demonstrate that a tumor CXCL9^Hi^ phenotype can be controlled through combinatorial nanotherapeutics to inflame the TME.

## 3 Discussion

Developing strategies to enhance immune cell trafficking to tumor sites is critical to improving immunotherapy responses. While the majority of current therapies have focused on T‐cell therapies,^[^ ^23^ ^]^ there is also a growing interest in myeloid cell therapies.^[^ ^24^, ^25^, ^26^, ^27^ ^]^ This renewed interest is driven in part by the realization and subsequent dissection of different myeloid cell subsets^[^ ^28^ ^]^ each with its own pro‐ and antitumor phenotypes.^[^ ^4^, ^29^ ^]^ A major interest has been the identification of abundant TAM subsets that correlate clinically with improved outcomes. One such subset is CXCL9^Hi^ expressing TAM^[^ ^8^, ^30^, ^31^ ^]^ and/or CXCL9^Hi^/SPP1^Lo^ ratio in TAM.^[^ ^4^, ^7^ ^]^ Based on this new understanding of biology, we set out to identify and develop translatable therapies that could pharmacologically mimic and enhance endogenous pathways of antitumor myeloid cells.

The expression of CXCL9, related chemokines (CXCL10), and other ISG is primarily driven by IFNGR signaling through IFNg produced by lymphocytes (e.g., through IL12, IL18, or antigen stimulation).^[^ ^32^, ^33^ ^]^ Within macrophages, IFNg response is mediated by JAK1/2, STAT1 signaling, which leads to transcription factor binding to gamma interferon activation site (GAS) elements and activation of IFNg programs in macrophages (Figure S11, Supporting Information). Additional pathways are being revealed by ongoing research. For example, in a recent study, CRISPR‐Cas9 screening identified numerous positive regulators of CXCL9, including Dnttip1, Prdm14, Zfp431, Klf6, Arid1a, Socs1 and Smarcd1.^[^ ^20^ ^]^ Interestingly, epigenetic regulation through the SWI/SNF‐PRC2 axis (e.g., by inhibiting EED or Irf1) led to up‐regulation of CXCL9. Thus, additional CXCL9 drug targets may become available in the future.

Here we identified a triple‐drug combination that works synergistically in upregulating CXCL9. The combination involves RBN2397, a PARP7 inhibitor in clinical trials, a STING prodrug (MSA‐2), and R848, a TLR 7/8 agonist. The ADP‐ribosyltransferase PARP7 modulates protein function by conjugating ADP‐ribose to the side chains of acceptor amino acids. PARP7 is expressed in various cells and can affect tumor growth through multiple mechanisms. For example, RBN2397‐mediated inhibition has been shown to induce both cancer cell‐autonomous effects and antitumor immunity via enhanced type I IFN signaling. It negatively regulates tank binding kinase 1 activity, which restrains phosphorylation and activation of the transcription factor IRF3,^[^ ^19^ ^]^ inhibiting androgen‐induced ADP‐ribosylation of the androgen receptor in prostate cancer^[^ ^34^ ^]^ and trapping PARP7 within the nucleus. MSA‐2 is an orally available non‐nucleotide STING agonist.^[^ ^35^ ^]^ Activation of STING by cyclic dinucleotide (CDN) ligands in human monocytes induces a type I IFN response and production of pro‐inflammatory cytokines associated with the induction of massive cell death.^[^ ^36^ ^]^ We show that a CANDI encapsulated prodrug formulation using prodrug MSA‐2p had similar effects in BMDM. Finally, R848 is a TLR7/8 agonist that activates the canonical NFkB pathway leading to IL12 secretion,^[^ ^37^ ^]^ which then stimulates T‐cells to produce IFNg.^[^ ^38^ ^]^ As we based our screening on the compounds’ ability to increase CXCL9 in IFNg‐exposed macrophages, this latter mechanism is important for triggering IFNg in vivo and maximizing the full efficacy of the compounds identified. Despite the different mechanisms of action of the small‐molecule modulators, the unifying theme was that they acted synergistically, presumably in part because they were delivered to TAM in an efficient manner. In prior work, it had been shown that up to 10% of injected dose/g tissue of CANDI partitions to tumors and where it is almost exclusively localized to myeloid cells.^[^ ^14^ ^]^ These works collectively emphasize the therapeutic potential of multiple pathway targeting for TAM re‐education in vivo.

Despite the design of the first efficient small‐molecule myeloid CXCL9 inducer system in this study, there were some limitations. First, to identify actionable hits rapidly, we quickly focused on the triple combination. Additional formulations, admixtures, and compounds may have similar or even stronger effects, and therapeutics beyond small‐molecules could activate CXCL9 phenotypes. Second, we started with a small library of compounds known to affect specific pathways. It is possible to perform unbiased screens of much larger libraries to identify new compounds with similar or even new mechanisms of action. Third, we show cellular uptake of CANDI400 in TAM, and prior work has quantitated tumor uptake.^[^ ^14^ ^]^ We show that ≈70% of all CD11b positive cells in tumors contain CANDI400, whereas tumor cells and lymphoid cells do not. It is theoretically possible to further increase TAM uptake by targeting ligands on CANDI400, an area that awaits exploration. For example, it is possible to attach ligands with TAM affinity, such as those that have been described in the literature.^[^ ^39^, ^40^, ^41^ ^]^ Fourth, while we showed initial proof of concept, additional work will be needed to ascertain and compare the effectiveness of CANDI400 in different types of cancers and combination therapies. It remains to be investigated how TAM‐targeted therapies influence tumor‐specific T‐cell responses and tumor control, and whether signals such as C/S ratio are key indicators of a tumor's immune “hot” or “cold” status. Despite these limitations, our initial results are extremely encouraging in developing a new class of myeloid cell‐directed therapeutics that capitalize upon the polarization of TAM toward CXCL9 to drive antitumor immunity.

## 4 Experimental Section

### Materials

All reagents and solvents were procured from Thermo Fisher or Sigma‐Aldrich and employed without further purification. Small‐molecules (Table S3, Supporting Information), namely 2‐Fucosyllactose, 2‐NP, Baicalein, Eganelisib, Entinostat, KIN‐1408, LCL161, MSA‐2, Picroside, Pidotimod, R848, Ruxolitinib, SR‐717, and Tilorone, were purchased from MedChemExpress; CRX527, MPLA, M‐TriDAP, and Murabutide were obtained from InvivoGen, while RBN2397 was acquired from AmBeed. The compounds were dissolved in dimethyl sulfoxide (DMSO) as appropriate and were utilized without further processing. MilliQ water was sourced from the Waters filtration system.

### Particle Synthesis

The synthesis of CANDI was further developed from a previously reported method.^[^ ^9^ ^]^ sbCD to produce smaller nanoparticles (17 nm vs 37 nm) are employed. sbCD de novo with a well‐defined degree of substitution (DS) of 2.5^[^ ^14^, ^42^ ^]^ addressing variability and high cost observed in commercially available products is synthesized. The resulting compound was then used to prepare CANDI nanoparticles, activated with EDC and NHS in MES buffer.^[^ ^14^ ^]^ Briefly, L‐lysine was added drop‐wise, and the reaction was allowed to stir for 18 h. The particles were precipitated with ice‐cold ethanol, purified, and characterized by DLS and Zeta potential before storage at −20 °C.

### Synthesis of MSA‐2 (4‐(5,6‐Dimethoxybenzo{b}Thiophen‐2‐yl)−4‐Oxobutanoic Acid)

The compound was synthesized using a previously published method, and the spectral data were consistent with this literature.^[^ ^43^ ^]^ Briefly, succinic anhydride (77 mg, 0.77 mmol, 3.0 equiv.) and AlCl_3_ (69 mg, 52 mmol, 2.0 equiv.) were dissolved in anhydrous DCM (3 mL) under argon and stirred at 0 °C for 30 min. Separately, 5,6‐dimethoxy benzothiophene (50 mg, 26 mmol, 1.0 equiv.) was dissolved in anhydrous DCM and added dropwise to the first mixture over 30 min. The reaction was stirred at 43 °C overnight. The dark green reaction was poured into ice water, and the pH was adjusted to ten using 1 M NaOH. The filtrate was collected and acidified to pH 2 using 1 µm HCL. The resulting brown precipitate was collected by gravity filtration, rinsed with water and DCM, dried, and collected to afford the desired product as a brown solid, 42 mg (55%). ^1^H NMR (400 MHz, DMSO‐D_6_) δ 12.20 (s, 1H), 8.21 (s, 1H), 7.60 (s, 1H), 7.48 (s, 1H), 3.86 (s, 3H), 3.84 (s, 3H), 3.26 (t, *J* = 6.4 Hz, 2H), 2.60 (t, *J* = 6.4 Hz, 2H). ^13^C NMR (101 MHz, DMSO‐D_6_) δ 192.5, 173.7, 150.8, 148.5, 140.6, 135.6, 132.5, 130.4, 106.6, 104.3, 55.9, 55.6, 33.1, 27.9. ESI‐MS for C_14_H_14_O_5_S {M‐H}‐: Calc'd: 293.05, Found: 293.27. {2M‐H}‐: Calc'd: 587.11, Found: 587.45.

### Synthesis of MSA‐2p (4a,7,8‐Trimethoxy‐2,3,4a,9b‐Tetrahydrodibenzo{b,d}Thiophene‐1,4‐Dione)

In an oven‐dried scintillation vial (20 mL) equipped with a magnetic stirrer have suspended a mixture of MSA‐2 (150 mg, mmol, equiv), and EDC (150 mg) in anhydrous acetonitrile: methanol mixture (3:1, 15 mL). The reaction was allowed to stir for 1 h at 25 °C and was monitored by LCMS. Upon full conversion to MSA‐2p, the solvents were evaporated and the crude was subjected to normal‐phase column chromatography (Hex: EtOAc 5%→40%). The pure fractions were collected and evaporated to yield MSA‐2p as a transparent oil that crystallized overnight under vacuum (67 mg, mmol, 43% yield). ^1^H NMR (400 MHz, DMSO‐D_6_) δ 7.55 (s, 1H), 7.40 (s, 1H, H1), 7.34 (s, 1H, H2), 3.82 (s, 3H, H3), 3.80 (s, 3H, H4), 3.23 (s, 3H, H5), 2.93–2.76 (m, 1H, H7&H8), 2.74–2.56 (m, 2H, H7, H8), 2.50 (m, 1H, H7, H8). ^13^C NMR (101 MHz, DMSO‐D_6_) δ 175.29, 148.52, 148.15, 139.33, 132.25, 131.84, 122.36, 107.92, 105.77, 104.51, 55.80, 55.61, 51.40, 35.84, 28.22. ESI‐MS for C_15_H_16_O_5_S {M‐H}^+^: Calc'd: 309.0718, found: {M‐H}^+^: 309.2200.

### Synthesis of 2D216

Following the well‐established amide coupling protocol previously reported,^[^ ^44^ ^]^ Compound 2D216 from activation of 4‐(Piperidine‐1‐sulfonyl)‐benzoic acid (50 mg, 0.19 mmol) using HATU (78 mg, 0.21 mmol) followed by amide formation with 4‐(2,5‐Dimethylphenyl)thiazol‐2‐ylamine (42 mg, 0.21 mmol) in the presence of triethylamine (28 mg, 0.28 mmol) is synthesized. Normal‐phase silica gel column chromatography yielded compound 2D216 (65 mg, yield = 77%) as an off‐white, pink solid. ^1^H NMR (400 MHz, CHLOROFORM‐d) δ 12.14 (br. s., 1H), 7.80 (d, *J* = 8.31 Hz, 2H), 7.63 (d, *J* = 8.56 Hz, 2H), 7.14 (s, 1H), 7.00 (s, 1H), 6.98 (d, *J* = 7.58 Hz, 1H), 6.87–6.93 (m, 1H), 2.93–3.01 (m, 3H), 2.29 (s, 3H), 2.24 (s, 2H), 1.61–1.68 (m, 4H), 1.39–1.46 (m, 2H). LCMS for C_23_H_26_N_3_O_3_S_2_ {M+H}+ calculated 456.13, found 456.71.

### Characterization—Particle Size

Particle size and surface charge for all nanoparticle formulations were determined by DLS and zeta potential measured on a Malvern Zetasizer APS at 5 mg mL^−1^ in PBS (0.5×) and 2 mg mL^−1^ in PBS (0.1×), respectively measured in DTS1170 cuvettes (Malvern) at 25 °C. All absorbance and fluorescent spectra (e.g, CANDI^AF647^ analogs) were performed with a multimode microplate reader (Tecan, Spark 500) using 96‐well transparent bottom black polystyrene microplates (Corning).

### Characterization—Small‐Molecule Loading of Nanoparticles

A solution of empty CANDI (CANDI^E^; 5 mg) in PBS (0.5×, 90 µL) was used for payload loading to a final DMSO concentration of 10%. The following nanoparticle compounds were prepared: CANDI400 containing MSA‐2p (0.26 mg), RBN2397 (0.1 mg), and R848 (0.22 mg). The solutions were vortexed rapidly until the complete dissolution of the drugs. All solutions were filtered through a 0.22 µm sterile filter (VWR) and used immediately for characterization, in vitro assays, or stored at −20 °C until further use *(Table* *S1* *, Supporting Information)*.

### Characterization—Turbidity Assay

CANDI^E^ stock solutions (2.5 mg mL^−1^, 0.5× PBS) were prepared at pH 7.4 and the small‐molecules were dissolved in DMSO (140 mM R848, 170 mM MSA‐2 Prodrug and 40 mM RBN2397) to prepare payload stocks. Loading of CANDI particles with payloads (0.1–52 mM, respectively, 10% DMSO) was quantified by absorbance scan measurement (λ_abs_ = 400–700 nm) after thoroughly mixing the solutions. Total loading of the payload was determined by the complete loss of absorbance. Data were normalized to payload‐free control and experiments were performed in triplicates (N = 3).

### Characterization—Loading Assessment by Nuclear Magnetic Resonance (NMR) and Liquid Chromatography‐Mass Spectrometry (LCMS)

NMR spectra were recorded on a Bruker Avance UltraShield 400 MHz spectrometer. ^1^H NMR chemical shifts were reported in ppm relative to SiMe_4_ (*δ* = 0) and were referenced internally concerning residual protons (*δ* = 4.79 for D_2_O). Peak assignments, calculated chemical shifts, and peak integrals were based on reference solvent peaks. 2D Rotating Frame Overhauser Enhancement Spectroscopy (2D‐ROESY) experiments were performed to assess the interactions between dipolarly coupled hydrogens, and integrals were normalized to the reference hydrogen (H^1^) of the sbCD. All experiments were performed in D_2_O (0.5 mL) at a fixed sCD concentration (26 mM) with 10% (CD_3_)_2_SO. High‐performance liquid chromatography‐mass spectrometry analysis (HPLCMS) was performed on a Waters instrument equipped with a Waters 2424 ELS Detector, Waters 2998 UV–Vis Diode array Detector, and a Waters 3100 Mass Detector. Separations employed an HPLC‐grade water/acetonitrile (0.1% formic acid) solvent gradient with XTerra MS C18 Column, 125 Å, 5 µm, 4.6 × 50 mm column; Waters XBridge BEH C18 Column, 130 Å, 3.5 µm, 4.6 × 50 mm.

### Characterization—MSA‐2p and MSA‐2 Loading Experiments

A stock of MSA‐2p (3.14 mg) was prepared in DMSO (70 µL). A solution of sbCD (64 mg in 500 µL water) was prepared. Turbidity experiments were performed in a 394‐well plate using 50 µL volume measuring absorbance point at 550 nm every ≈100 s for 5 cycles with 60 s shaking between cycles. The average values from the individual cycles gave the best approximation of the state of the turbid suspensions. Using a fixed concentration of 0.13 mg per well, ranges of sbCD concentration equalling 0, 0.224, 0.448, 0.896, 1.79, 3.58, and 7.16 equivalents of MSA‐2p are titrated. To obtain the curve for MSA‐2 an initial concentration stock of 3.0 mg in 70 µL DMSO was used. All points were averages of three independent replicates (*n* = 3).

### Characterization—MSA‐2p Hydrolysis Experiments

A stock of MSA‐2p (3 mg) in DMSO (750 µL) was prepared for hydrolysis experiments in phosphate aqueous buffers (pH 4–8) at a 10% concentration. A solution of sbCD (6–36 mg in 800 µL buffer) was prepared and mixed with the MSA‐2p stock (90%). All experiments were performed by sequential reading of an absorbance scan between 300–500 nm, a single point at 325 nm followed by 5 s quick mixing to ensure homogeneous mixing. Each experiment was performed in triplicates in time lapses of 85 cycles (2000 s). The percent of MSA‐2p converting to MSA‐2 (%) was calculated by correlating the total absorbance value at 325 nm to the relative MSA‐2 to MSA‐2p concentration quantified as the area under each peak by LCMS (Figure S5, Supporting Information).

### Characterization—Drug Release Kinetics

Kinetics of drug release were performed in a closed dialysis set‐up employing a 3 kDa molecular weight cut‐off membrane (Pur‐A‐Lyzer Midi Dialysis Kit). Solutions of the empty nanoparticle CANDI^E^ (50 mg) were loaded with R848 (2.2 mg), MSA‐2p (2.6 mg), and RBN2397 (1.0 mg) in PBS (1×, 1 mL) containing 10% DMSO and were dialyzed against PBS (1×, 5 mL) at 37 °C under constant stirring (600 rpm). The percentage of eluted molecules was quantified by analysis of the liquid chromatographs at different time points (t = 0, 1, 5, 10, 25, 45, 60, 90, 120, 180, 210, 240, 270, 300, 330, 360, 390 min), injecting a total of 90 µL aliquots into an LC‐MS and subsequently replacing the system with additional 100 µL of PBS. Each payload was identified by its unique retention time (R848 = 0.90 min, MSA‐2 = 1.57 min, RBN2397 = 1.87 min) and mass‐to‐charge ratio (R848 = 314 {M}^+^ ES^+^, MSA‐2 = 293 {M}^−^ ES^−^, RBN2397 = 522 {M}^−^ ES^−^ MSA‐2 Prodrug = 309 {M}^+^ ES^+^). The cumulative drug release was determined as the ratio of the integrated area under the curve for each eluted peak to the total area under the curve of chromatographs obtained from the non‐membrane controls. All experiments were performed in triplicates (N = 3).

### Characterization—Transmission Electron Microscopy

CANDI400 particles were freshly prepared (50 mg mL^−1^, PBS 1×) and diluted with water to a final concentration of 0.1 mg mL^−1^. The particle solution was charged on a TEM grid for 1 min and treated with a 2% aqueous uranyl acetate solution for 15 min, followed by three washing steps with ultra‐pure water (×3). Imaging was performed in a transmission electron microscope (JEOL 2100).

### Characterization—Nanoparticle Tracking Analysis (NTA)

A stock of pharmaceutically loaded CANDI400 was prepared in freshly filtered PBS (50 mg mL^−1^). Next, the total particle count, size distribution, and homogeneity of four dilutions (×62.5, ×125, ×250, ×500, N = 3) are estimated. The particle counts obtained from each concentration were multiplied by the dilution factor and averaged to obtain the total particle count depicted in Figure S6 (Supporting Information). All experiments and analyses were performed using a Panalytical NanoSight LN10 (Malvern) nanoparticle characterization system. All nanoparticle tracking analyses (NTA) were done with identical experiment settings.

### In Vitro Experiments—Immortalized Cell Lines

The immortalized murine bone marrow‐derived macrophages (iMACs)^[^ ^45^ ^]^ were acquired from Charles L. Evavold (Ragon Institute, Harvard University) and used to assess toxicity (Table S4, Supporting Information). Briefly, iMAC cells were plated and grown in Dulbecco's Modified Eagle Medium (DMEM, Corning) supplemented with 10% Fetal Bovine Serum (FBS, Corning) and 1% Penicillin Streptomycin (Corning) at 37 °C and 5% CO_2_ and MC38 cells were cultured in Iscove's Modification of DMEM (Corning). Upon reaching confluency, cells were split using 0.05% Trypsin / 0.53 mM EDTA (Corning), and all in vitro assays were performed after the cells reached 90% confluency. Prior to cell culture application, all CANDI preparations were filtered through a 0.22 µm sterile filter (VWR).

### In Vitro Experiments—Bone Marrow‐Derived Cells

Murine BMDC were isolated from CXCL9‐RFP/CXCL10‐BFP reporter mice, IL12‐eYFP reporter mice, or wild‐type C57BL/6J mice. BMDC of reporter mice were employed for flow cytometry and live‐cell microscopy analyses, while BMDC of wild‐type cells were utilized to evaluate cytokine induction. To obtain the whole bone marrow, femurs were prepared and flushed with sterile PBS using syringes and a 28‐gauge needle. RBC Lysis Buffer (BioLegend) was then used according to the manufacturer's instructions to lyse red blood cells. The remaining cells were counted using a Neubauer chamber and seeded into either transparent (NEST, flow cytometry analysis) or black (ibidi, glass bottom for imaging) 96 well plates at a density of 1.25 × 10^5^ cells per well. For cytokine assays, cells were seeded into transparent 6‐well plates (Corning) at a density of 1 × 10^6^ cells per well. BMDM were differentiated by adding 50 ng mL^−1^ recombinant murine M‐CSF (BioLegend) to cell culture media for 7 days. New media was added every 3–4 days.

### In Vitro Experiments—Cytokine Screen

To determine the effect of Nanoparticle drug loading on broader cytokine induction, wild‐type C57BL/6J BMDM were seeded and stimulated with 50 ng mL^−1^ IFNg. Subsequently, CANDI400 formulation (5 µg mL^−1^) or empty control nanoparticles were added for 24 h. The conditioned media were then collected for cytokine array analysis. Cytokine array analysis was performed using the Proteome Profiler Mouse Cytokine Array Kit, Panel A (R&D, ARY006) according to the manufacturer's instructions. Images of the membranes were obtained (Azure Sapphire Biomolecular Imager) and quantified using ImageJ.

### In Vitro Experiments—Toxicity

iMACs were seeded in 96 well plates at a density of 15 × 10^3^ cells per well and incubated for 24 h at 37 °C and 5% CO_2_ before use. Stock solutions of different CANDI nanoparticles (Table S1, Supporting Information) were prepared and then diluted in cell culture medium to desired concentrations (0.09 µg mL^−1^ to 10 mg mL^−1^, DMSO 0.5%). Cells were incubated for 2.5 h with nanoparticles before the medium was exchanged. Cells were further incubated for 48 h at 37 °C and 5% CO_2_ before adding MTT (3‐(4,5‐dimethylthiazol‐2‐yl)−2,5‐diphenyl‐2H‐tetrazolium bromide) solution (5 g L**^−1^** in FluoroBrite DMEM, 10% final) to each well. After 3 h, the supernatant was carefully removed, and metabolized formazan was dissolved with isopropyl alcohol. Plates were shaken at 500 rpm on a microplate shaker (VWR) for 30 min, and the absorbance of each well was measured (λ_abs_ = 550 nm). Triplicates were obtained for each concentration tested, and IC_50_ values were calculated from means.

### In Vitro Experiments—Flow Cytometry

was used to assess cytokine induction and characterize the TME. BMDM of CXCL9‐RFP/CXCL10‐BFP reporter mice or IL12‐eYFP reporter mice were stimulated o/n with the respective drug combinations, then trypsinized and washed with PBS. Next, the cells were stained using AquaAmine LiveDead Fixable viability stain (Thermo Fisher) diluted in PBS, followed by treatment with Fc block (BioLegend) and fluorochrome‐conjugated antibodies (Tables S2 and S3, Supporting Information) diluted in FACS buffer (1x PBS, 2 mM EDTA, 2% FBS). For flow cytometry measurements, cells were resuspended in a FACS buffer. All conditions were measured in triplicates in Attune NxT flow cytometer (Thermo Fisher), and the data was analyzed using FlowJo 10 software (TreeStar).

### In Vitro Experiments—Dose‐Response

To determine the dose‐response of the triple labeled nanoparticle, a stock solution of CANDI400 nanoparticles (Table S1, Supporting Information) was prepared and subsequently diluted in cell culture medium to achieve the desired concentrations (0.08 pg mL^−1^ to 1 mg mL^−1^, DMSO 0.5%). CXCL9‐RFP/CXCL10‐BFP BMDM from reporter cells were incubated overnight in the nanoparticle‐spiked media before removing the media. Cells were then prepared for microscopy and flow cytometry analysis.

### In Vitro Experiments—Live‐cell microscopy

was performed to determine the cytokine production in BMDM of reporter mice (Table S5, Supporting Information) with or without IFNg stimulation. Harvested cells were treated with various combinations of small‐molecules (0–10 µm, DMSO <0.5%) for 24 h by adding prepared stock solutions to cell culture media. Cells were imaged in a 96‐well plate. Before imaging, cells were stained with Hoechst 33 342 (15 µg mL^−1^, Thermo Fisher) or SYTO 11 Green Fluorescent Nucleic Acid Stain (2.5 µµ, Thermo Fisher) according to the manufacturer's protocol. Fluorescence microscopy was performed using an IX81 inverted fluorescence microscope (Olympus, Tokyo, Japan) equipped with a motorized stage (Renishaw, Wotton‐under‐Edge, England, UK) and fitted with an ORCA‐Fusion Digital CMOS camera (Hamamatsu Photonics, Hamamatsu, Japan). Using CellSens Dimension 3.1.1 software (Olympus), multiple fields of view were acquired for each sample with a UPlanSApo ×10 (numerical aperture (NA) 0.75, Olympus) or a UPlanSApo ×40 air objective (NA 0.95, Olympus). In addition to brightfield, four fluorescent channels were acquired DAPI (345/455 nm), GFP (489/508 nm), YFP (550/565 nm), CY3 (550/565 nm), and CY5 (625/670 nm) were excited with the appropriate optical filters.

### In Vivo Experiments—Mouse Models

All animals were bred and housed under specific pathogen‐free conditions at the Massachusetts General Hospital. Experiments were approved by the MGH Institutional Animal Care and Use Committee (IACUC) and were performed in accordance with MGH IACUC regulations. CXCL9‐RFP/CXCL10‐BFP mice (N = 9) were employed for the assessment of CXCL9 induction. IL12p40‐eYFP mice (N = 2) were used for IL12 induction experiments. Female C57BL/6J mice (N = 18) were utilized for MC38 tumor growth experiments (Figure S10; Table S5).

### In Vivo Experiments—Intravital Microscopy

Mice‐bearing dorsal window chambers with MC38‐mTAG‐GFP were performed to determine the kinetics of CXCL9 induction in the TME (Figure 5 and Figure S8, Supporting Information). Dorsal window chambers were implanted into Rex3 mice using well‐established techniques.^[^ ^46^ ^]^ Fluorescent tumor cells (MC38‐H2B‐GFP) were implanted in the window chambers as previously described^[^ ^47^, ^48^ ^]^ and allowed to grow for 7–21 days before imaging experiments, with tumor growth monitored regularly. In additional experiments, the vascular half‐life of CANDI400 by serial imaging of the microvascular in the mouse ear were determined(Figure S9, Supporting Information).

All confocal images were collected using a customized Olympus FV1000 confocal microscope (Olympus America). A 2x (XLFluor, NA 0.14), a 4x (UPlanSApo, NA 0.16), and an XLUMPlanFL N 20x (NA 1.0) water immersion objective were used for imaging (Olympus America). Fusion‐protein CXCL10‐BFP, tumor cells (MC38‐H2B‐GFP) and SYTO 11 Green, fusion‐protein CXCL9‐RFP, and CANDI^AF647^ were excited sequentially using a 405, a 473, a 559, and a 633 nm diode laser in combination with a DM‐405/488/559/635 nm dichroic beam splitter. Emitted light was further separated by beam splitters (SDM‐473, SDM‐560, and SDM‐640) and emission filters BA430‐455, BA490‐540, BA575‐620, and BA655‐755 (Olympus America). Confocal laser power settings were carefully optimized to avoid photobleaching, phototoxicity, or tissue damage. Fiji (ImageJ, 2.9.0/1.53t) was used for image analysis.

### In Vivo Experiments—Drug Treatment

CANDI400 was administered by tail‐vein injection (100 µL PBS 0.5x, pH 7.4) containing 5 mg nanoparticle (0.22 mg of R848, 0.26 mg of MSA‐2 Prodrug, 0.10 mg of RBN2397). Before injection, the solution was sterilized by filtration through a 0.22 µm sterile centrifugal filter (VWR), vortexed, and used promptly or frozen at −20 °C.

### Statistical Analysis

All statistical data analyses were performed using GraphPad Prism 9 software, and results were expressed as mean ± standard deviation. A 2‐tailed Student's t‐test and one‐way ANOVA followed by Bonferroni's multiple comparison tests for normally distributed datasets are used. Non‐parametric Mann‐Whitney or Kuskal‐Wallis tests when variables are not normally distributed are performed. For survival analysis, *p* values were computed using the Log Rank test. *p* values > 0.05 were considered insignificant (n.s.), and *p* values < 0.05 were considered significant. ^∗^ *p* value < 0.05, ^∗∗^ *p* value < 0.01, ^∗∗∗^ *p* value < 0.001, ^∗∗∗∗^ *p* value < 0.0001.

## Conflict of interest

RW has consulted for ModeRNA, Boston Scientific, Lumicell, Seer Biosciences, Earli, and Accure Health, none of whom contributed to this research. MJP has consulted for AstraZeneca, Elstar Therapeutics, ImmuneOncia, KSQ Therapeutics, Merck, Siamab Therapeutics, Third Rock Ventures, and Tidal. The other authors report no affiliations.

## Author Contributions

N.E. and E.A.H. contributed equally to this work. M.P. and R.W. performed conceptualization. N.E., E.A.H., I.F., and R.K. performed data acquisition. All authors performed data analysis. K.S. and E.A.H. performed synthesis. M.P., C.G., and R.W. performed supervision. R.W. wrote and visualized the original draft. R.W. and all coauthors wrote, reviewed, and edited. R.W. performed funding acquisition, project administration and resources.

## Supporting information

Supporting information

## Data Availability

The data that support the findings of this study are available from the corresponding author upon reasonable request.
