# Supplementary material for: Pharmacological Polarization of Tumor‐Associated Macrophages Toward a CXCL9 Antitumor Phenotype
Source: Adv Sci (Weinh). 2024 Feb 11;11(15):2309026. doi: 10.1002/advs.202309026 (PMC11022742; doi:10.1002/advs.202309026)
Supplement: Supplementary file 1 — Supporting information [file ADVS-11-2309026-s001.pdf]

ADVANCED SCIENCE
Open Access

Supporting Information

for *Adv. Sci.*, DOI 10.1002/advs.202309026

Pharmacological Polarization of Tumor-Associated Macrophages Toward a CXCL9 Antitumor Phenotype

*Noah Enbergs, Elias A. Halabi, Anne-Gaëlle Goubet, Kelton Schleyer, Ina R. Fredrich, Rainer H. Kohler, Christopher S. Garris, Mikaël J. Pittet and Ralph Weissleder**

Supporting information

Pharmacological polarization of tumor-associated macrophages towards a CXCL9 anti-tumor phenotype

Noah Enbergs[1#], Elias A. Halabi[1#], Anne-Gaëlle Goubet[2,3], Kelton Schleyer[1], Ina R. Fredrich[1], Rainer Kohler[1], Christopher S. Garris[1], Mikaël J. Pittet[2,3,4], Ralph Weissleder[1,5,*]

[1] Center for Systems Biology, Massachusetts General Hospital, 185 Cambridge St, CPZN 5206, Boston, MA 02114

[2] Department of Pathology and Immunology, University of Geneva, Geneva, Switzerland

[3] AGORA Cancer Research Center, Swiss Cancer Center Leman, Lausanne, Switzerland

[4] Ludwig Institute for Cancer Research, Lausanne, Switzerland

[5] Department of Systems Biology, Harvard Medical School, 200 Longwood Ave, Boston, MA 02115

# equal contributions

*R. Weissleder, MD, PhD
Center for Systems Biology
Massachusetts General Hospital
185 Cambridge St, CPZN 5206
Boston, MA, 02114
617-726-8226
rweissleder@mgh.harvard.edu

Supplementary Figures

Figure S1: Screening approach and in vitro drug effects. CXCL9-RFP/(Rex3) reporter mice were used to harvest bone marrow cells. Cells were cultured with MCSF for seven days to yield bone marrow-derived macrophages (BMDM). These cells were then exposed to different drugs and drug combinations for 24 hrs, and RFP was measured by microscopy. This resulted in several hits, including the triple combination of RBN2397, MSA-2, and R848, which were then loaded into the 16 nm cyclodextrin NP drug delivery system. **B**. In order to determine whether cytokine production in BMDM was specific to the Nanoparticle delivery system, we compared empty and triple drug-loaded nanoparticle. Note the exclusive cytokine (ISG pattern) production with triple-loaded CANDI400.

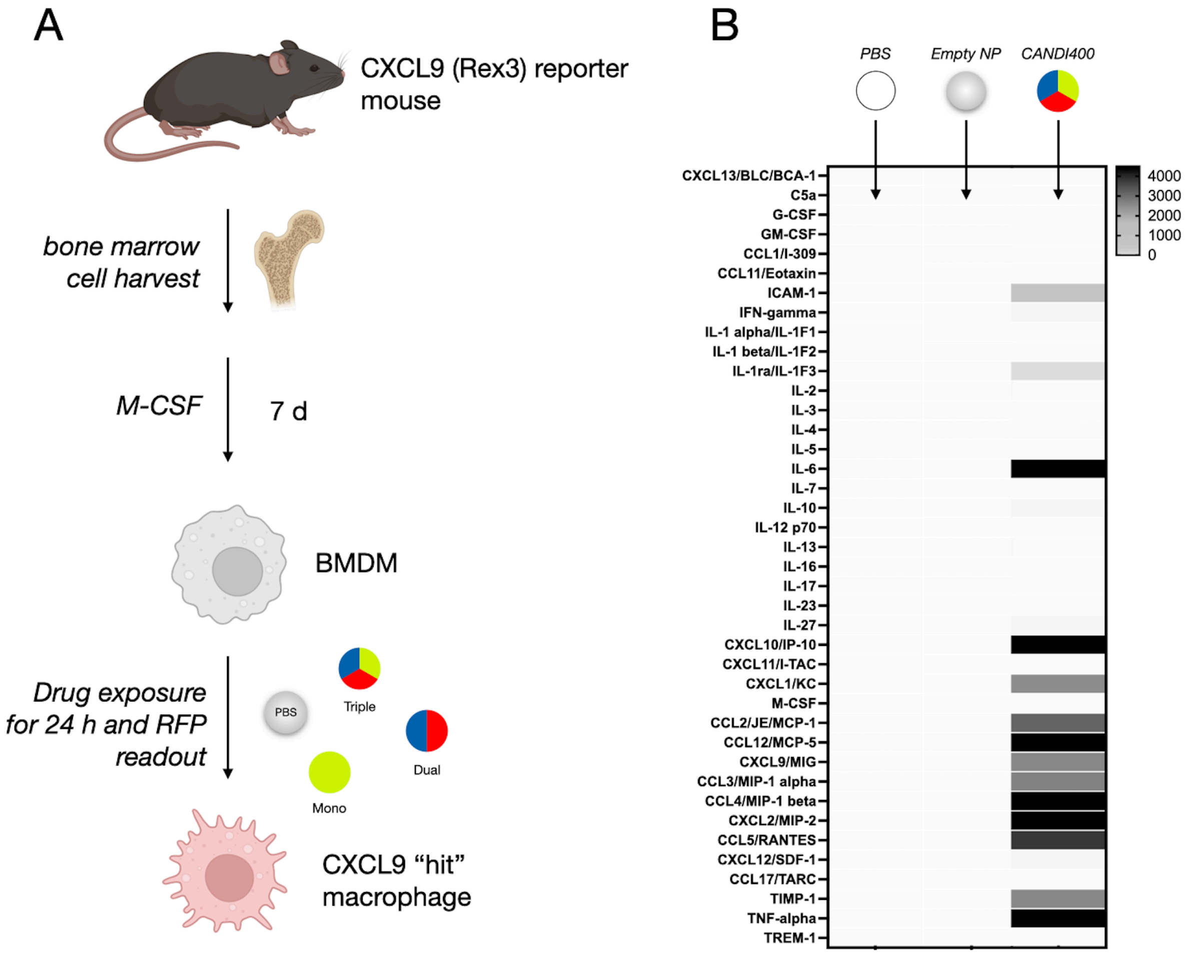

Figure S2: Synthesis of 2D216. A. Synthesis of TLR4 agonist 2D216. **B.** 1H-NMR spectrum of 2D216 in $CDCl_3$. Protons are assigned based on two-dimensional COSY NMR (data not shown). **C**. HPL-chromatogram of purified compound using a reverse-phase mobile phase with a retention time of ~1.7 min for 2D216. **D**. Absorbance spectrum of pure 2D216 depicting absorbance maxima at 250 and 312 nm. **E.** Mass chromatograms of 2D216 correspond to the calculated exact mass in the negative and positive modes.

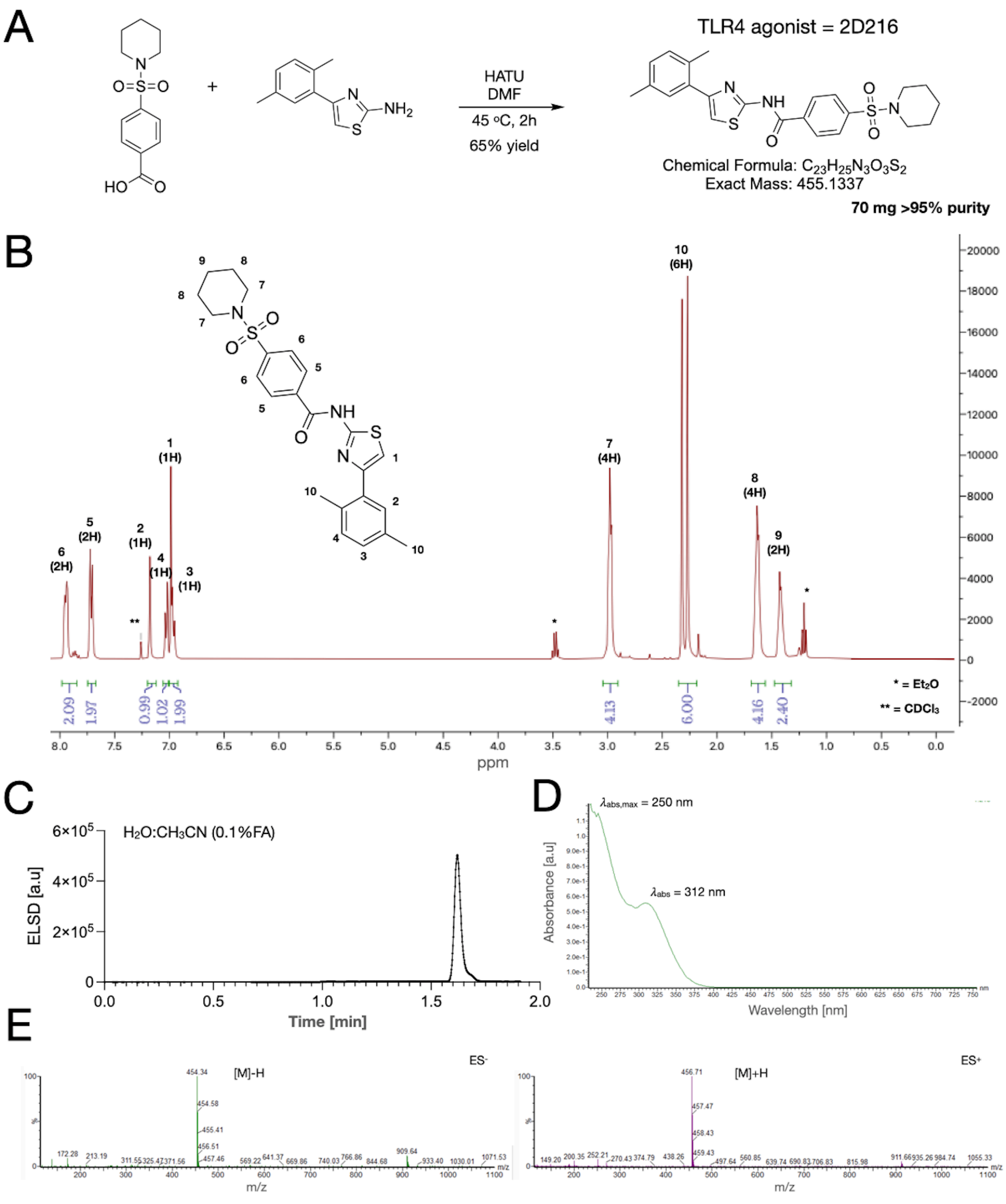

Figure S3: Characterization of MSA-2 (panel A) and MSA-2p (panel B) by NMR deuterated DMSO-D$_6$. ^{1}H-NMR spectra are shown on the left. The assigned protons were determined based on two-dimensional COSY, HMBC and HSQC NMR analysis (data not shown). The respective ^{13}C-NMR spectra of each molecule are shown on the right.

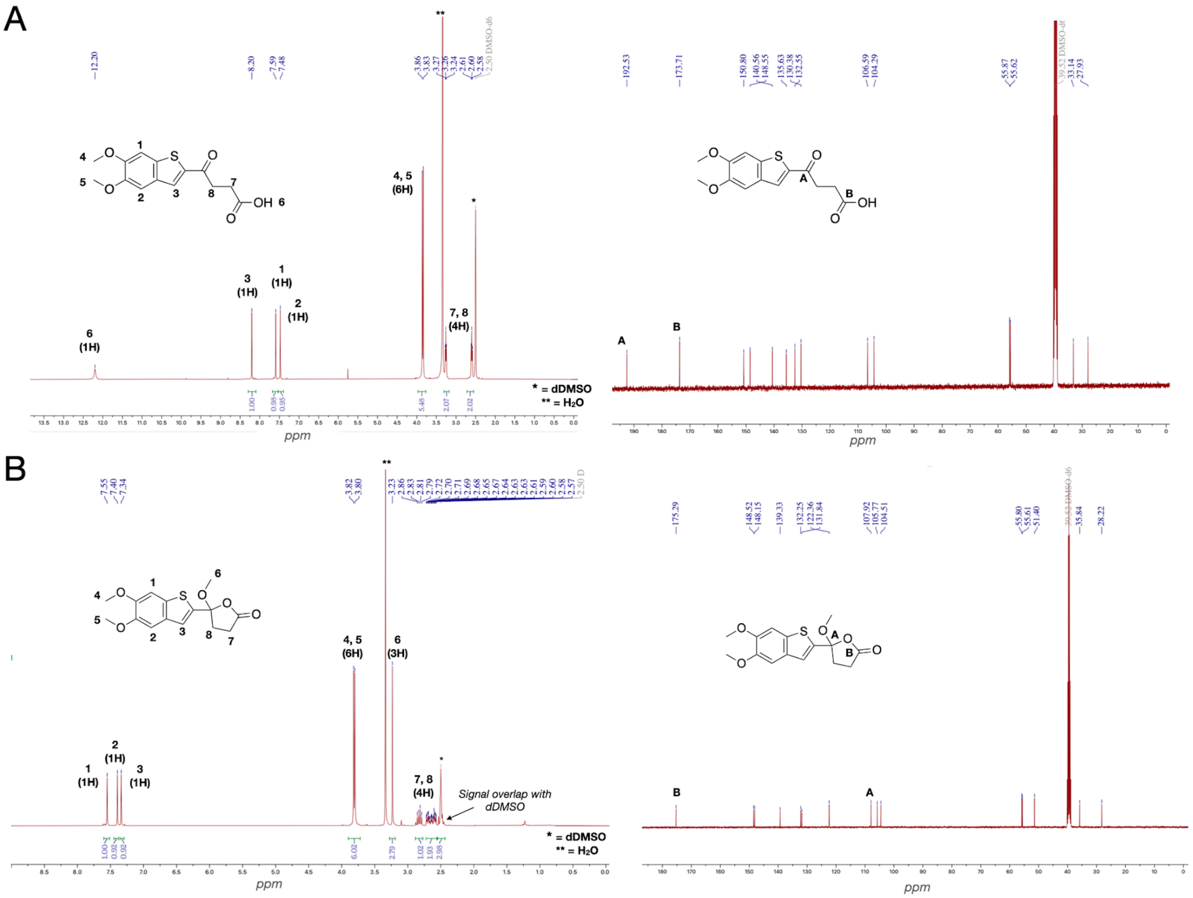

Figure S4: NMR titration experiments to provide evidence of inclusion complexation with **A.** R848 and **B.** RBN2397. The total concentration of sbCD per condition was kept constant (10 mg, 500 µL D_2O) and a variable concentration of the guest molecules R848 (0.3→2 equiv.) or RBN2397 (0.1→2) were added in DMSO-D_6. For R848 (panel A), we observed the protons in the aromatic groups of the molecule (7.0–8.5 ppm) undergoing large changes in chemical shifts (zoomed-in region on the left). Additionally, the inner cavity protons of the sbCD also underwent considerable changes in chemical shift (2.5–2.7 ppm). For RBN2397 (panel **B**), we noticed a decreased in solubility of the molecule in >0.5 equiv. compared to sbCD. This indicates that RBN2397 requires at least 3.5 eq of sbCD to be fully soluble.

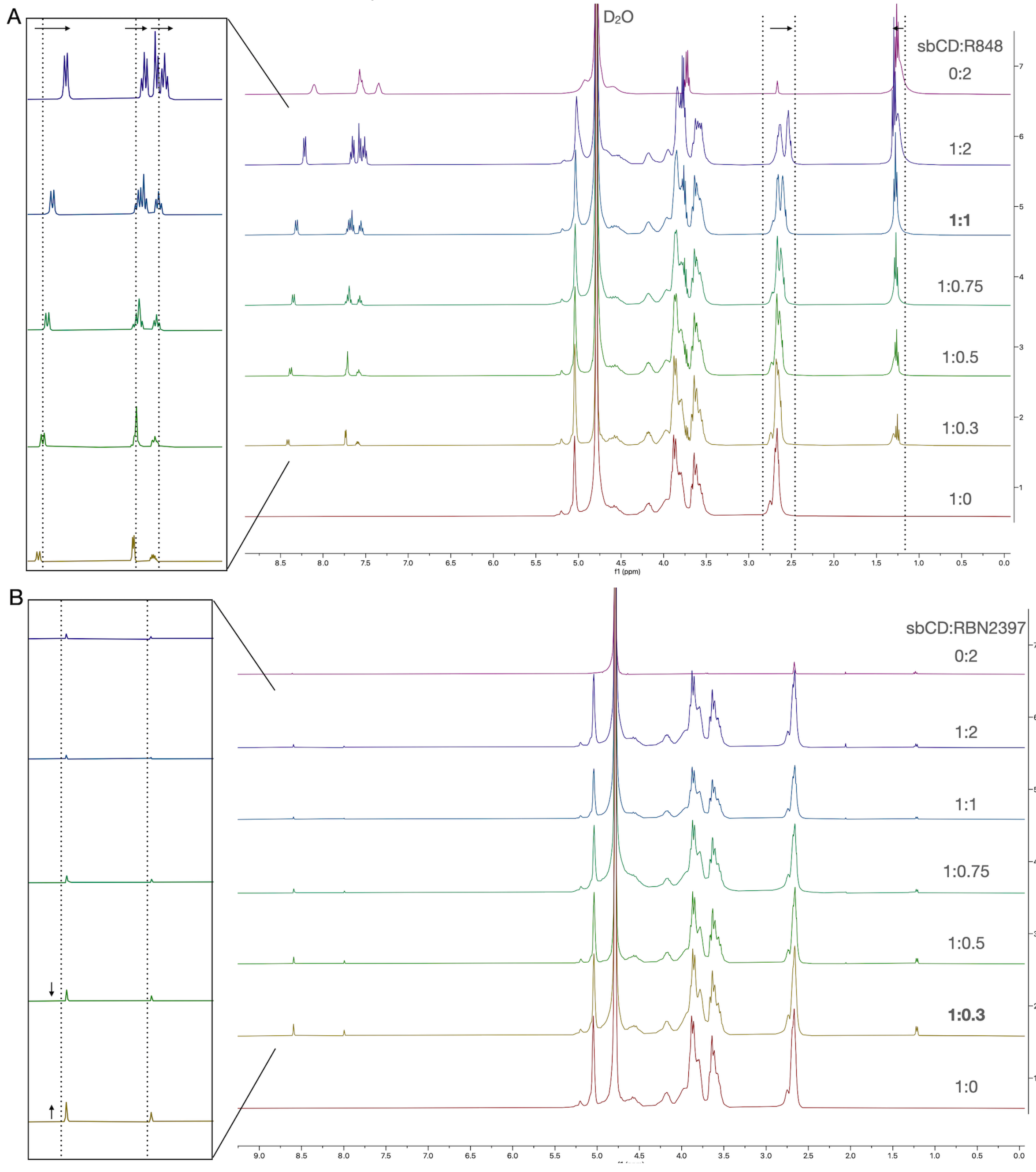

Figure S5: Mechanism of Hydrolysis of MSA-2p. A. Schematic depiction of the proposed hydrolysis mechanism starting from the release of the methoxy group and subsequent attack by hydroxide ion to open the cyclic product and yield MSA-2. **B.** UV-Vis spectra comparing MSA-2 to MSA-2p. From this graph, it is evident that the loss of the second carbonyl functional group in MSA-2p, creates a hypsochromic shift in the absorbance maxima, resulting in a significant loss of absorbance at λ = 325 nm. **C.** Hydrolysis experiments of MSA-2p at pH 4.5 and 7.4 (top) indicate almost full conversion to MSA-2 after 500 s by LCMS (bottom). **D.** Hydrolysis experiments for MSA-2p at pH 7.4 using increasing amounts of sbCD indicated as (equiv.) to MSA-2p (top). We noticed that increasing the amount of sbCD decreased the rate of hydrolysis of MSA-2p, which was optimal at a neutral pH (bottom). **E.** Liquid chromatograph showing the quantitative conversion of MSA-2p to MSA-2 of the data shown in panel **D** (top). **F.** Mass chromatograms of MSA-2p and MSA-2 in the positive (ES^+) and negative (ES^-) mode, respectively, showing the presence of the parent compound. All hydrolysis experiments were performed in triplicates (N = 3) and monitored at 325 nm wavelength.

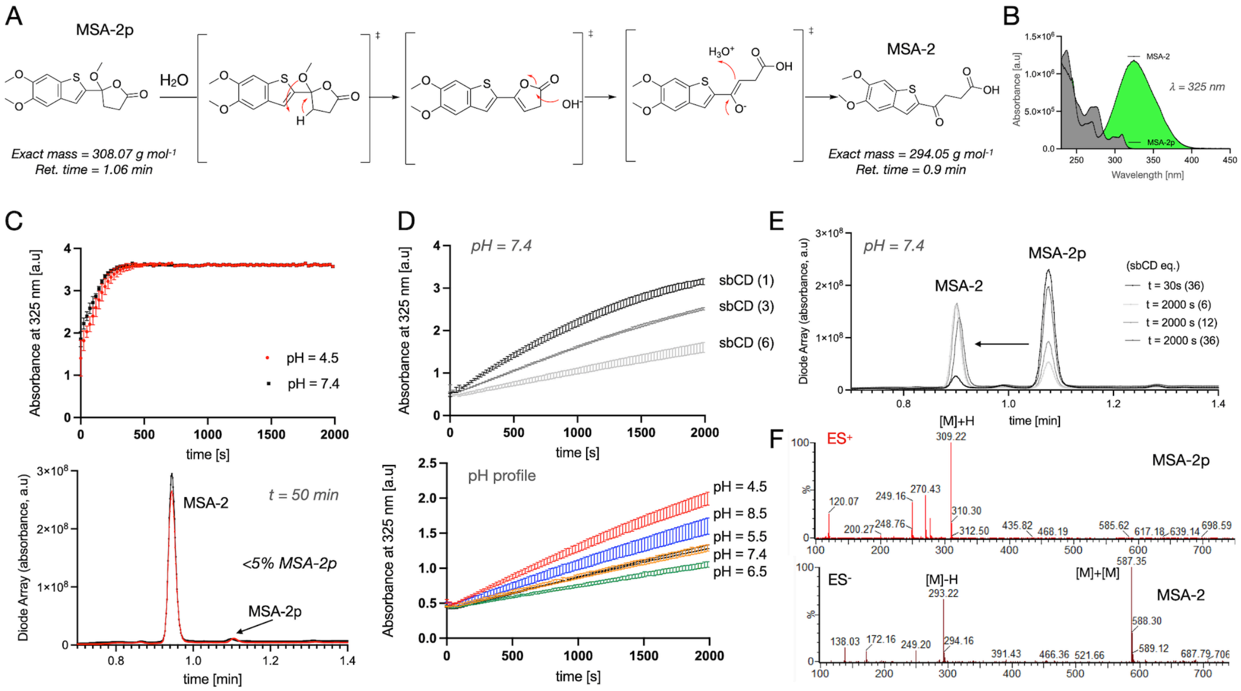

Figure S6: Formulation of small molecule hits into TAM-avid nanoparticle (CANDI400). A. Dynamic light scattering (DLS) of empty and triple-loaded CANDI400. Drug-loaded formulation contained MSA-2 prodrug (0.26 mg), RBN2397 (0.1 mg), and R848 (0.22 mg) per 5 mg of the particle without noticeable changes in the nanoparticle size or polydispersity index (PDI). **B**. Zeta potential measurements of the surface charge of the empty vs. loaded CANDI400 suggest that the surface of the particles undergoes an approximate +5 mV change upon complexation of the triple combo. **C.** A standard curve showing the calculated nanoparticles per mL using Nanoparticle Tracking analysis (NTA) obtained from testing four concentrations of CANDI400 (N = 3). The in vivo dose was thus 1.23×10^{11} ml^{-1} particles. **D**. Release kinetics of triple small molecules from CANDI400 using a porous membrane (3 kDa) in PBS (1x) at 37 ºC. The cumulative release rates for R848, MSA-2, and RBN2397 are depicted as individual replicates (N = 3). The dissociation rates (k_{off}) and complex half-life ($t_{1/2}$) were determined and compared as averaged from the 7 h time-lapse quantification.

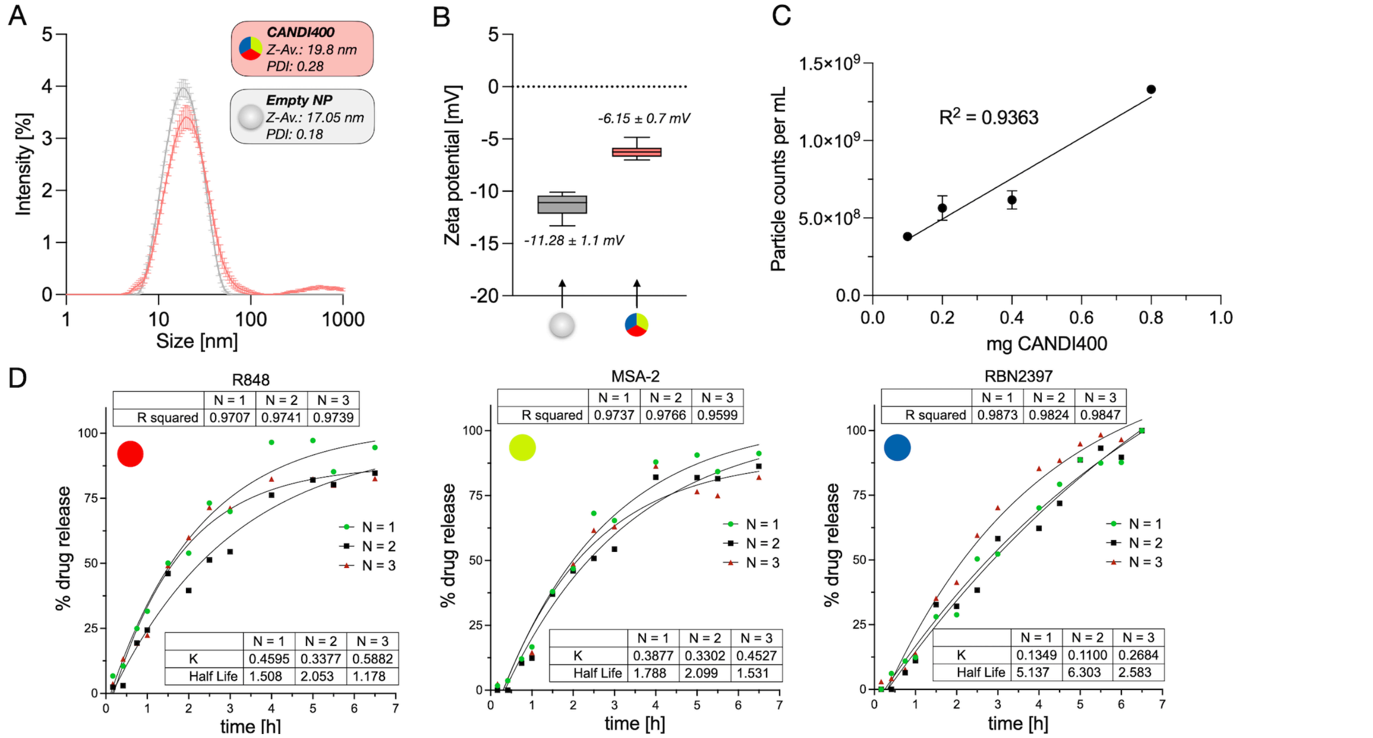

Figure S7: Microscopy images from screens. Representative fluorescence images of bone marrow-derived macrophages from different donor mice (see **Table 5**), following incubation with small molecules. All images were windowed and leveled identically to show differences in induction. **A**. Images showing CXCL9-RFP (red, 550 nm channel) induction under different treatment conditions (50 ms exposure time, all images are windowed and leveled the same way for comparison). The highest CXCL9 induction was seen with the triple combination of RBN2397+MSA-2+R848 (Bottom left in yellow). **B**. Identical set-up as in A but showing CXCL10 expression (cyan, 405 nm channel). The highest CXCL10 expression was again seen with the triple combination. **C.** Images showing IL12-eYFP induction (yellow, 488 nm channel) with different treatments. Scale bar = 200 μm.

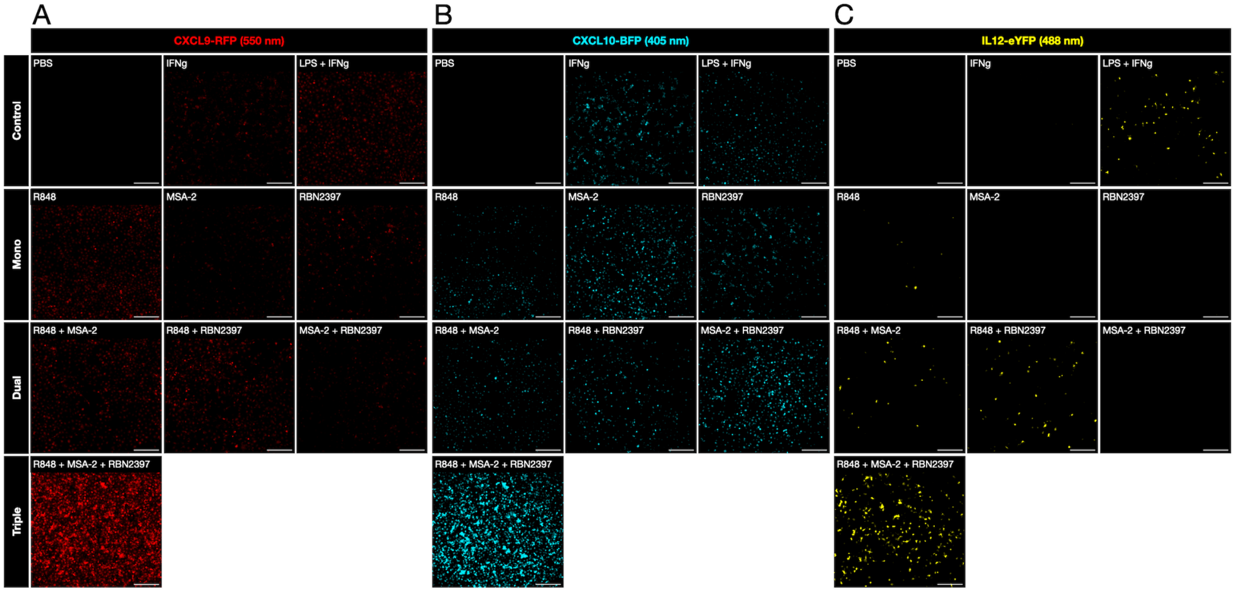

Figure S8: Intravital microscopy of CXCL9 induction in the MC38 mouse model. 40x magnification of tumor microenvironment in a live mouse with an MC38-H2B-GFP tumor (green, 473 nm channel). Note the CANDI400 (white, 633 nm channel) accumulation in non-tumor cells (macrophages) within 24 hrs after IV administration. All CANDI400-containing cells have high CXCL9-RFP expression (red, 559 nm channel). CXCL10-BFP (blue, 405 nm channel) expression co-localizes with CXCL9. Scale bar = 20 μm.

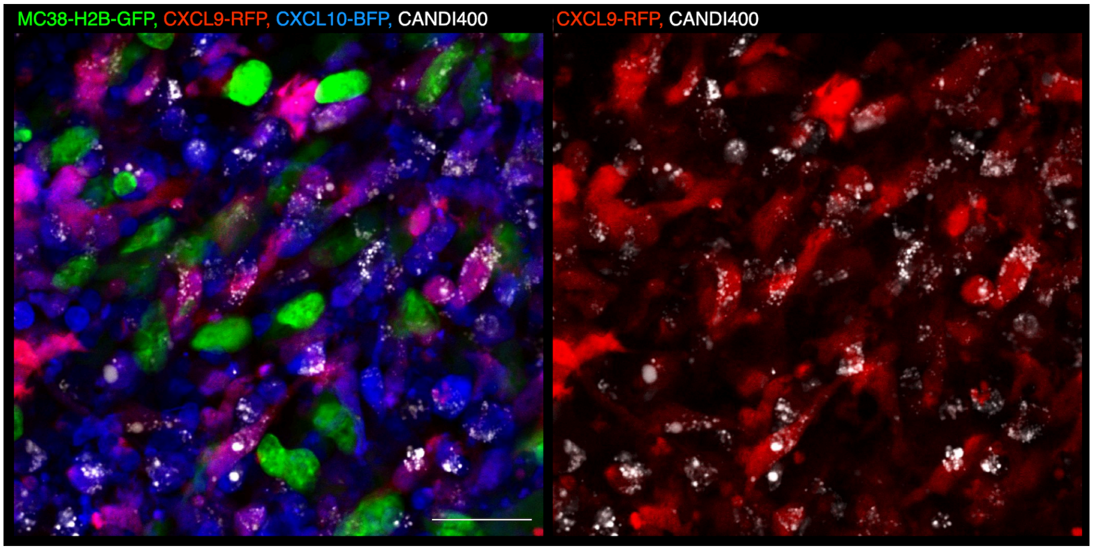

Figure S9: Vascular half-life. Intravital imaging of the intact mouse ear following IV administration of CANDI400. The graph plots the vascular signal intensity as a function of time. Fitting of the data points revealed a bi-exponential decay with a minor fast half-life of 0.09 hrs and a major half-life of 2.3 hrs. R^2=0.008. Note the cellular uptake becoming visible at the 1-4 h time points. Similar kinetics were observed in the tumor microenvironment.

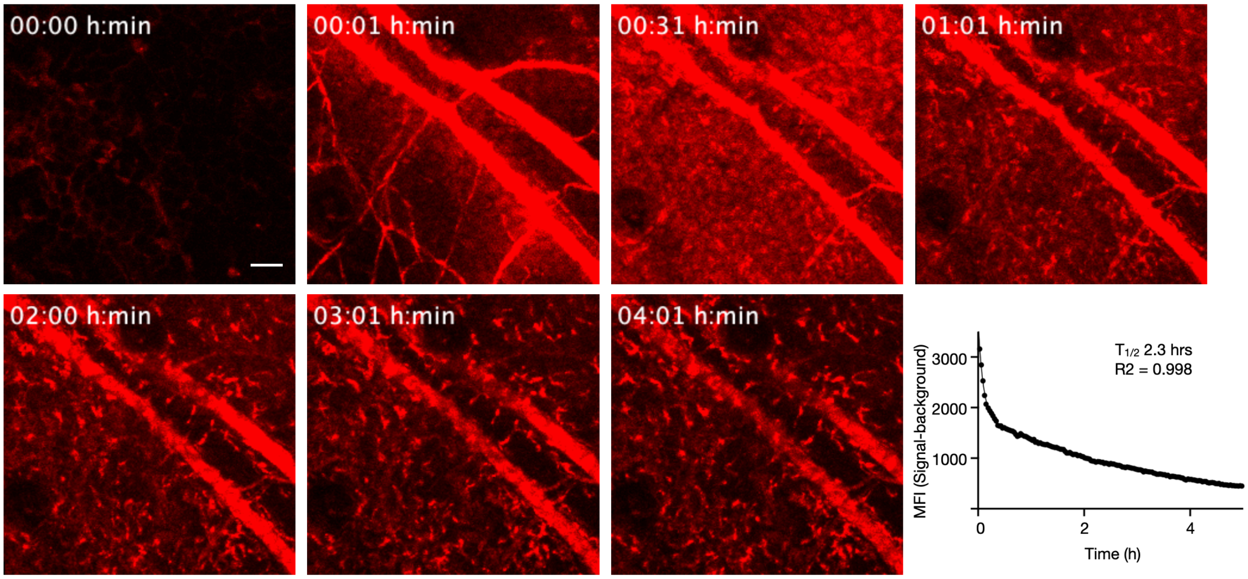

Figure S10: In vivo efficacy in MC38 tumor model. A. Mice (N = 18) were implanted with MC38 tumors eight days prior to IV administration of CANDI400. Note the anti-tumor efficacy of CANDI400 in this model. **B**. Representative images of the inoculation site at day 20. Note the complete absence of visible tumor in a representative CANDI400 treated mouse.

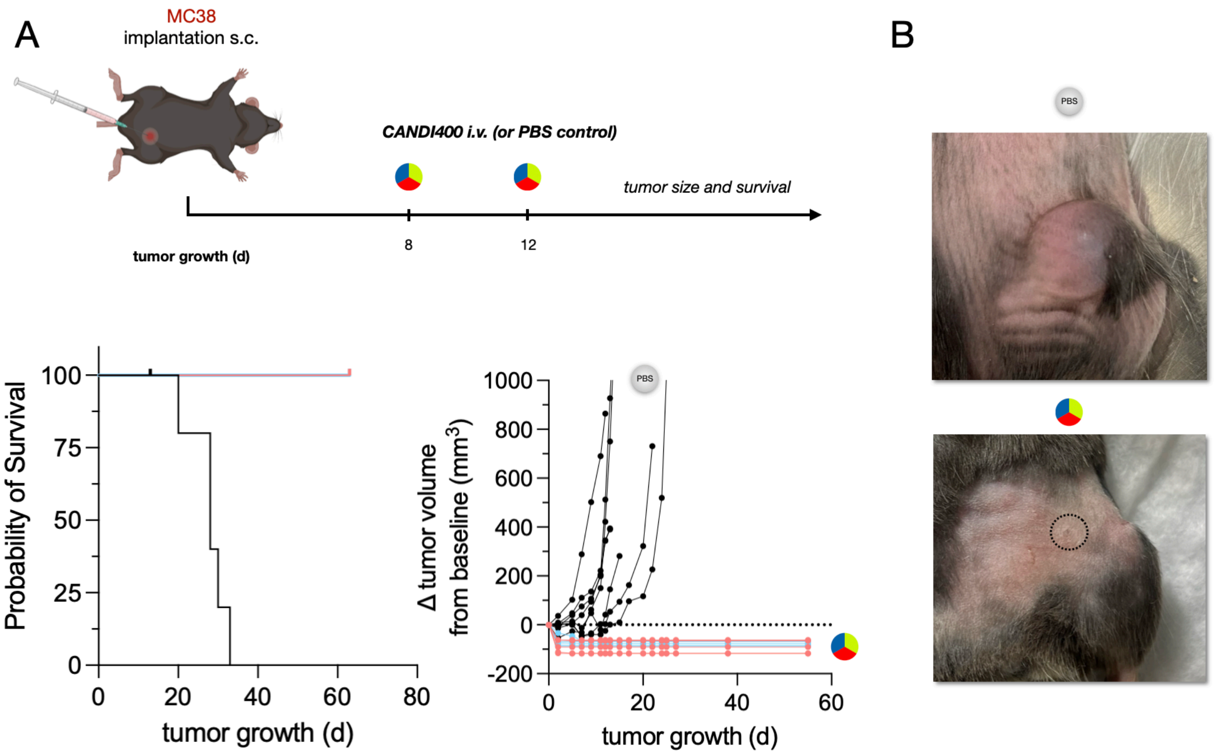

Figure S11: Overview of TAM drug actions. In the study, we targeted multiple TAM pathways by co-delivering small molecule antagonists and agonists. Together, multi pathway modulation resulted in robust drug effects that were synergistic and led to supra-physiological up-regulation of CXCL-9 and other interferon-stimulated gene (ISG) products (IL12, among others). This, in turn, results in the recruitment of effector T cells in the tumor environment and "licenses" them via IL12 production to become more effective ICI drug targets.[38] There may be additional direct tumor effects via CANDI400 stimulated myeloid cells (TNFa, boosting antigen presentation in DC, phagocytosis, and others).

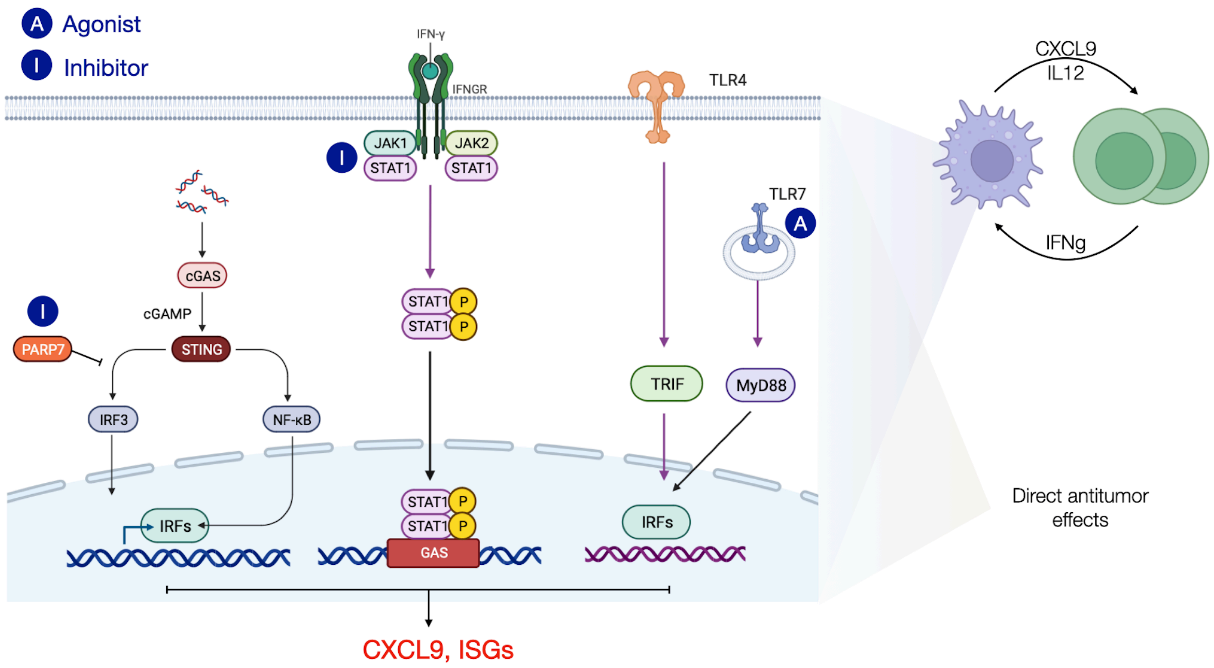

Table S1. Overview of nanoparticle properties with respective characterization by dynamic light scattering (DLS, average size in nm), polydispersity index (PDI). All experiments were performed as triplicates of triplicates (N = 9).

Name	Code	Payload	Drug loading per mg of CANDI	Size (nm)	PDI
CANDI[E]	CANDI100	None	NA	16.7±2.7	0.251
CANDI400	CANDI400	RBN2397+MSA-2 +R848	0.118	19.8±1.1	0.28

Table S2. List of antibodies used for flow cytometry and immunohistochemistry.

Markers	Target population	Source	Dye	Catalog #	Clone
Anti-Mouse CD11b	Myeloid cells	BioLegend	PE/Cy5	101210	M1/70
Anti-Mouse CD80	Myeloid cells	Invitrogen	APC	17-0801-81	16-10A1
Anti-Mouse CD86	Myeloid cells	BioLegend	BV605	105037	GL-1
Anti-Mouse F4/80	Myeloid cells	Invitrogen	PE	12-4801-82	BM8
LIVE/DEAD	dead cells	ThermoFisher	fixable aqua	L34957	
Anti-Mouse CD19	B-Cells	BioLegend	PE/Cy5	152405	1D3/CD19
Anti-Mouse CD45.1	Hematopoetic Cells	BioLegend	AF700	110724	A20
Anti-Mouse C8a	CD8 T-Cells	BD Bioscience	APC/Cy7	557654	53-6.7
Anti-Mouse MHC-II	APCs	BioLegend	Pacific Blue	107620	M5/114.15.2
Anti-Mouse CD90.2	T-Cells	BioLegend	BV605	140318	53-2.1
Anti-Mouse CD11b	Myeloid cells	BioLegend	BV650	101259	M1/70
Anti-Mouse CD11c	Myeloid cells	BioLegend	BV711	117349	N418
Anti-Mouse CD4	CD4 T-Cells	BioLegend	BV785	100552	RM4-5
Anti-Mouse NK1.1	NK Cells	BioLegend	PE	108708	PK136

Table S3. List of drugs used for mini library.

Drug	Molecular target	Source	Catalog #
2D216	TLR agonist enhancer	Synthesized	NA
2-Fucosyllactose	Immunomodulator	MedChemExpress	HY-N9965
2-NP	IFNg enhancer	MedChemExpress	HY-W013523
Baicalein	PI3Kγ inhibitor	MedChemExpress	HY-N0196
CRX527	TLR4 agonist	InvivoGen	Tlrl-crx527
Eganelisib	PI3Kγ inhibitor	MedChemExpress	HY-100716
Entinostat	HDAC inhibitor	MedChemExpress	HY-12163
KIN-1408	RLR agonist	MedChemExpress	HY-19961
LCL161	cIAP inhibitor	MedChemExpress	HY-15518
MPLA	TLR4 agonist	InvivoGen	tlrl-mpls
MSA-2	STING agonist	MedChemExpress	HY-136927
M-TriDAP	NOD2 agonist	InvivoGen	tlrl-mtd
Murabutide	NOD2 agonist	InvivoGen	tlrl-mbt
Picroside	STAT inhibitor	MedChemExpress	HY-N0407
Pidotimod	Immunoregulatory Modifier	MedChemExpress	HY-B0944
R848	TLR7/8 agonist	MedChemExpress	HY-13740
RBN2397	PARP7 inhibitor	AmBeed	2381037-82-5
Ruxolitinib	JAK1 inihibitor	MedChemExpress	HY-50856
SR717	STING agonist	MedChemExpress	HY-131454
Tilorone	IFNg agonist	MedChemExpress	HY-B1080

Table S4. Cell lines.

Cell Line	Manufacturer / obtained from	Catalog Number	Use
BMDM	CXCL9-donor mice	NA	Screen
MC38-H2B-GFP	Chiara Cianciaruso	RRID:CVCL_B288	Tumor growth
iMACs	Charles L. Evavold	NA	toxicity

Table S5. Mouse strains.

Name	Manufacturer	Catalog Number	N
CXCL9-RFP/CXCL10-BFP	Andrew Luster, MGH	PMID: 23123063	N = 9
B6.129-IL12btm1Lky/J	Jackson Laboratory	Stock# 006412 RRID:IMSR_JAX:006412	N = 2
C57BL/6J	Jackson Laboratory	Stock# 000664 RRID:IMSR_JAX:000664	N = 18

References

[38] C. S. Garris, S. P. Arlauckas, R. H. Kohler, M. P. Trefny, S. Garren, C. Piot, C. Engblom, C. Pfirschke, M. Siwicki, J. Gungabeesoon, G. J. Freeman, S. E. Warren, S. Ong, E. Browning, C. G. Twitty, R. H. Pierce, M. H. Le, A. P. Algazi, A. I. Daud, S. I. Pai, A. Zippelius, R. Weissleder, M. J. Pittet. *Immunity* **2018**, *49*, 1148.
